# CULTURAL AND SOCIAL RESILIENCE FACTORS ON HEALTH IN THE CONTEXT OF IMMIGRATION

**DOI:** 10.1093/geroni/igz038.116

**Published:** 2019-11-08

**Authors:** XinQi Dong, Mengting Li, Man Guo

**Affiliations:** 1 Rutgers Institute for Health, Health Care Policy and Aging Research, New Brunswick, United States; 2 Rutgers, The State University of New Jersey, New Brunswick, New Jersey, United States; 3 University of Iowa, Iowa, United States

## Abstract

Acculturation is a process whereby immigrants change their beliefs or behaviors in response to the prevailing norms and values in the host country. Acculturation may directly affect health outcomes, while it also operates through multi-level social factors, such as family relations, social network, and neighborhood cohesion, in shaping immigrants’ health. Asian Americans are the fastest growing minority group in the United States. Chinese Americans constitute the largest segment of Asian Americans. The five studies aim to profile multi-level cultural and social resilience factors of older Asian Americans’ health by analyzing the Asian American Quality of Life survey and the Population Study of Chinese Elderly in Chicago (PINE). Two studies, Acculturation and Cognitive Health and Factors Associated with Unmet Healthcare Needs demonstrated the direct effect of acculturation on health. Another two studies outlined a more complex mechanism between cultural and social determinants and health. Perceived Stress, Social Support, and Dry Mouth found the buffering effect of social support on the relationship between perceived stress and oral health. Neighborhood Social Integration, Social Network, and Cognitive Function identified micro- and macro-level resilience factors exert interaction effects on cognitive function. In addition, previous studies pay little attention to the dynamic nature of social relations. Transition in Family Relations in Immigrant Families took a typology approach to capture multifaceted family relations, with a longitudinal design to explore the transitions in family relations in the process of acculturation and its impact on mental health. This symposium will build an integrative resilience model for older Asian Americans.

